# Targeting MCH Neuroendocrine Circuit in Lateral Hypothalamus to Protect Against Skeletal Senescence

**DOI:** 10.1002/advs.202309951

**Published:** 2024-09-25

**Authors:** Bin Guo, Yong Zhu, Shuai Lu, Xiangming Chen, Zhuoqun Ren, Yuqi Liu, Hao Luo, Chao Wang, Xucheng Yang, Jianxi Zhu

**Affiliations:** ^1^ Department of Orthopaedics Xiangya Hospital Central South University Changsha 410008 China; ^2^ Department of Orthopedic Trauma Beijing Jishuitan Hospital Capital Medical University Beijing 100035 China; ^3^ Beijing Research Institute of Traumatology and Orthopaedics Beijing 100035 China; ^4^ Xiangya School of Medicine Central South University Changsha 410008 China; ^5^ National Clinical Research Center for Geriatric Disorders Xiangya Hospital Central South University Hunan 410008 China; ^6^ Hunan Key Laboratory of Aging Biology Xiangya Hospital Central South University Hunan 410008 China

**Keywords:** BMSC, lateral hypothalamus, melanin concentrating hormone, neuroendocrinology, osteogenesis, skeletal senescence

## Abstract

Neuroendocrine regulation is essential for maintaining metabolic homeostasis. However, whether neuroendocrine pathway influence bone metabolism and skeletal senescence is unelucidated. Here, a central neuroendocrine circuit is identified that directly controls osteogenesis. Using virus based tracing, this study is identified that melanin concentrating hormone (MCH) expressing neurons in the lateral hypothalamus (LH) are connected to the bone. Chemogenetic activation of MCH neurons in the LH induces osteogenesis, whereas inhibiting these neurons reduces osteogenesis. Meanwhile, MCH is released into the circulation upon chemogenetic activation of these neurons. Single cell sequencing reveals that blocking MCH neurons in the LH diminishes osteogenic differentiation of bone marrow stromal cells (BMSCs) and induces senescence. Mechanistically, MCH promotes BMSC differentiation by activating MCHR1 via PKA signaling, and activating MCHR1 by MCH agonists attenuate skeletal senescence in mice. By elucidating a brain‐bone connection that autonomously enhances osteogenesis, these findings uncover the neuroendocrinological mechanisms governing bone mass regulation and protect against skeletal senescence.

## Graphical abstract

This study reveals a new neuroendocrine circuit connecting the lateral hypothalamus to bone, where MCH neurons enhance bone formation and protect against skeletal aging. Activating these neurons increases MCH levels, promoting bone marrow stromal cell differentiation and osteogenesis via MCHR1‐PKA signaling, suggesting potential therapeutic targets for osteoporosis and skeletal senescence.

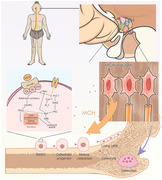

## 1 Introduction

Bone undergoes continuous dynamic metabolism, which is traditionally thought to be regulated by secretion of humoral hormones.^[^ ^1^ ^]^ In senile or pathophysiological conditions, disruption of bone metabolic homeostasis leads to the development of osteoporosis, which is a common marker for skeletal senescence.^[^ ^2^ ^]^ Although various therapeutic interventions have been developed to alleviate osteoporosis, including intermittent parathyroid hormone (PTH), Vitamin D, bisphosphonates, and monoclonal antibodies, a complete cure for osteoporosis is still lacking due to limited understanding of the key regulatory factors of osteogenesis. Recent evidence suggests that the brain has a crucial role in the delicate regulation of bone metabolism. The bone is robustly innervated by sensory nerve which transmits signal to the brain.^[^ ^3^ ^]^ The brain can sense the status of bone metabolism through skeletal interception, providing feedback and monitoring the internal state of the skeleton to regulate osteogenesis.^[^ ^4^ ^]^ In skeletal interoception, bone mass is sensed through nonconscious mechanisms, like the sensing of blood glucose fluctuations and gut motility.^[^ ^5^ ^]^ Several studies have revealed the significant role of the sympathetic nervous system in bone metabolism.^[^ ^6^ ^]^ It regulates both bone formation and resorption by influencing osteoblastic and osteoclastic cells.^[^ ^7^ ^]^ Hormonal interactions, particularly involving leptin, are also key to this process.^[^ ^4^, ^8^ ^]^ The balance and timing of sympathetic activity are crucial for maintaining bone health. Additionally, research in microgravity conditions offers insights into the adaptability of bone metabolism in different environments.^[^ ^9^ ^]^ Overall, the sympathetic nervous system's role in bone health is complex, involving hormonal, cellular, and environmental interactions. However, the mechanisms by which the neuroendocrinological circuitry from bone to the brain controlling bone metabolism remain unclear.

The hypothalamus acts as a central control center, transmitting information from the forebrain to regulate whole‐body metabolism and maintain homeostasis. By orchestrating the autonomic nervous system and regulating hormonal activity, the hypothalamus plays a dominant role in the maintenance of metabolic homeostasis, including body temperature, thirst, hunger, and sleep‐wake cycles.^[^ ^10^ ^]^ The lateral hypothalamus (LH), a crucial part of the hypothalamus, governs energy metabolism including feeding and sleeping by secreting hormones like orexin and melanin concentrating hormone (MCH).^[^ ^11^ ^]^ Interestingly, orexin has been found to regulate skeletal homeostasis and bone mass through the activation of orexin receptor 2 (OX2R) in the LH, promoting bone formation^[^ ^12^ ^]^ and reversing bone loss in hypoxia.^[^ ^13^ ^]^ However, the function and effect of MCH on bone mass regulation have not been elucidated.^[^ ^12^, ^14^ ^]^ Additionally, the hypothalamic action of leptin and ghrelin, have been found to regulate bone metabolism by affecting sympathetic tone. Neuropeptide Y (NPY), secreted by the hypothalamus, has also been implicated to regulate bone formation through central actions.^[^ ^15^ ^]^ However, the neuroendocrinological circuit that integrates skeletal interoception and the molecular mechanisms of these hormones on bone tissue aside from sympathetic system remain largely unknown.^[^ ^5^ ^]^

To address this issue, we initially employed virus‐based tracing^[^ ^16^ ^]^ to confirm that MCH neurons in LH, but not orexin neurons, are directly connected to bone. We observed that the activity of MCH neurons in LH exhibited a strong correlation with bone metabolic status. Specifically, the activity of MCH neurons in LH was significantly reduced in cases of osteoporosis. Furthermore, plasma concentration of MCH was significantly lowered in mice with osteoporosis. To further investigate the function of MCH neurons in LH in the regulation of bone mass, we utilized chemogenetic viruses and transgenic mouse models to manipulate this specific group of neurons. Chemogenetic stimulation of MCH neurons in LH resulted in robust osteogenesis and accelerated bone tissue regeneration. Single cell analysis revealed that BMSC senescence was aggravated in chemogenetic inhibition of MCH neurons in LH. Subsequent in vitro experiments demonstrated the expression of MCHR1 in BMSCs and revealed that MCH induces osteogenesis through the activation of MCHR1 and the PKA pathway. Collectively, by elucidating this novel neuroendocrinological pathway, our study uncovers the role of MCH neurons in LH in sensing bone metabolism and controlling osteogenesis by secreting MCH to stimulate BMSC differentiation. Targeting MCH pathway in LH could be an effectively therapeutic intervention for skeletal senescence.

## 2 Results

### 2.1 Bone is Connected to MCH Neurons in LH

**Figure 1:**
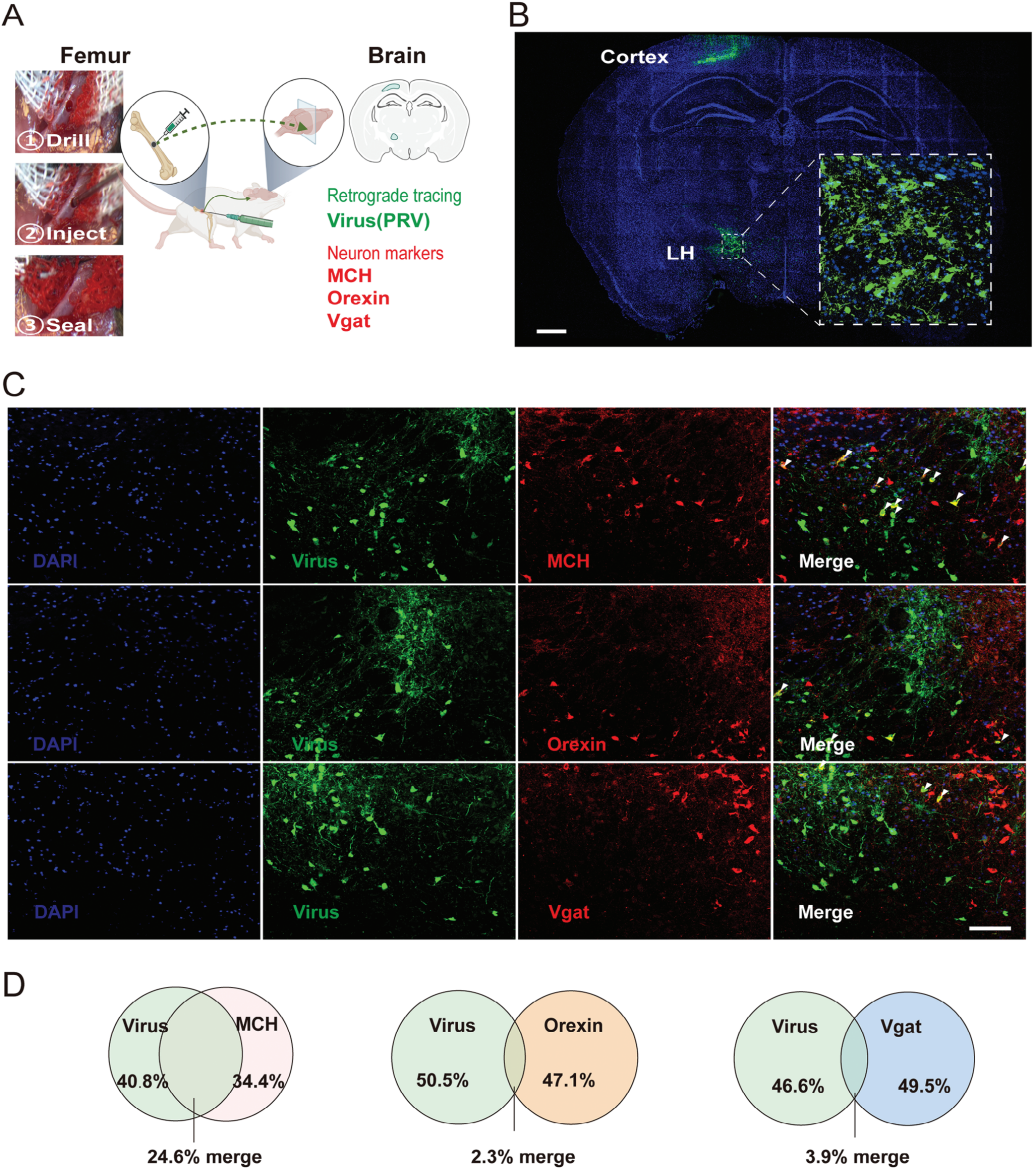
Bone is connected to MCH neurons in LH. A) Schematic of the injection method for anterograde tracing of the bone. B) Representative image showing HSV co‐stained with MCH. C) Representative image showing HSV co‐stained with MCH (upper), Orexin (middle), and Vgat (lower) in the LH. D) The Venn diagram showing the overlap between HSV fluorescence and MCH (left), Orexin (middle), and Vgat (right) in the LH.

To elucidate the central neuronal populations implicated in bone mass regulation, we first identified neurons with direct connections to bone. We utilized a novel approach involving the injection of Herpes Simplex Virus (HSV‐129), engineered to encode Enhanced Green Fluorescent Protein (EGFP), directly into the bone marrow of adult male mice (**Figure** **1**A). This technique enabled us to anterogradely and trans‐synaptically label central neurons receiving bone‐derived signals. Seven days post‐injection, consistent observation revealed a discrete cluster of EGFP‐positive neurons within a specific region of the lateral hypothalamus, precisely located between −2.00 mm bregma, ±1.37 mm lateral to the midline, and at a depth of −4.65 mm (Figure 1B).

Further investigations were conducted to determine the specific subtypes of neurons directly connected to bone. Co‐staining of these EGFP‐positive neurons in the LH with major protein markers including Melanin‐Concentrating Hormone (MCH), orexin, and Vesicular Glutamate Transporter 2 (Vglut2) was performed. Remarkably, the majority of EGFP‐positive neurons demonstrated co‐localization with MCH (based on all fluorescence signals, all fluorescence signals include virus green fluorescence and neuron marker red fluorescence. The yellow fluorescence generated by the superposition of the two in the cell (Figure 1C). The percentage of yellow fluorescence in all fluorescence signals is the standard (Figure 1D), and the overlap of MCH, Orexin, Vgate with the virus is confirmed to be 24.5%, 2.3%, and 3.9%, respectively. In contrast, only a minimal percentage of these neurons exhibited co‐staining with orexin or Vglut2. These findings collectively indicate a trans‐synaptic connection between bone and a specific population of MCH neurons in the lateral hypothalamus, indicating a novel neural pathway in bone mass regulation.

### 2.2 Activity of MCH Neurons in LH Correlates with Osteogenesis

**Figure 2:**
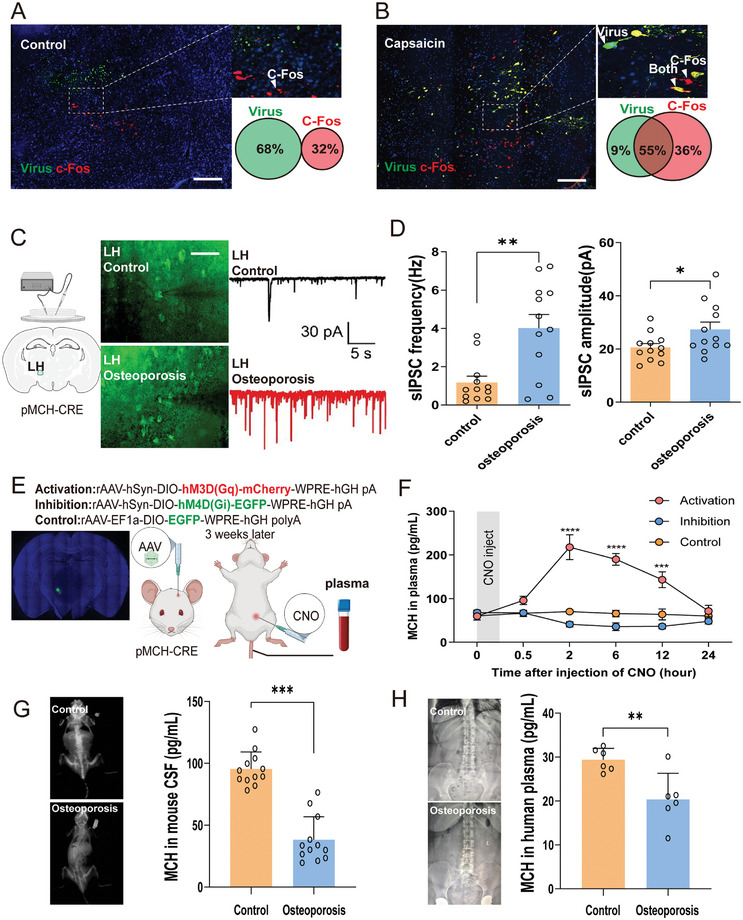
Activity of MCH neurons in LH correlates with osteogenesis. A) Representative image and the Venn diagram showing the merge of HSV and c‐fos positive neurons in LH in control state (n = 3). B) Representative image and the Venn diagram showing the merge of HSV and c‐fos positive neurons in LH 4 h after capsaicin stimulation. C) Representative sIPSCs of MCH neurons in LH in control (upper) and osteoporotic (lower) groups. D)The sIPSC frequency is increased in osteoporosis group. control: n = 12 from 3 mice, p = 0.0015, t (22) = 3.620 compared with osteoporosis group (n = 12 from 3 mice), unpaired t test. Quantitative analyses of the sIPSC amplitude from 2 groups. control: n = 12 from 3 mice, p = 0.0409, t (22) = 2.173 compared with osteoporosis group (n = 12 from 3 mice), unpaired t test. ^*^ *p* < 0.05, ^**^ *p* < 0.01. E) Schematic of the injection method for chemogenetic virus in LH and injection of CNO. F) Concentrations of MCH in plasma after injection of CNO in chemogenetic activation, inhibition, and control group (n = 7). G) Concentrations of MCH in mouse CSF of osteoporosis mice and normal controls (n = 12). H) Concentrations of MCH in human plasma of osteoporosis patients and normal controls (n = 6).

To explore whether the activity MCH neurons in LH are connected to the bone and correlates with osteogenesis, we first used c‐Fos (an immediate early gene upon neuronal stimulation) staining to examine the rapid activation of MCH neurons in LH upon bone stimulation as previously described.^[^ ^17^ ^]^ Capsaicin or saline was used in bone marrow to rapidly stimulate central reaction. If the HSV positive cell were also c‐Fos positive, they were regarded to be responsible for bone stimulation. 4 h after injection, the brain sliced were harvested for c‐Fos staining. We found that the capsaicin stimulation group had a significantly higher c‐Fos and HSV double positive cell count compared with control group (**Figure** **2**A,B). There were nearly no HSV and c‐Fos double positive cells before capsaicin stimulation. However, 55% cells were HSV c‐Fos positive 4 h after capsaicin stimulation. To further examine the activities of MCH neurons in LH at different bone mass, we used age induced osteoporotic mice to record the spontaneous inhibitory postsynaptic currents (sIPSCs) of MCH neurons in LH (Figure 2C) by patch clamp. Since the sIPSCs refer to the flow of ions across the postsynaptic membrane that leads to a decrease in spontaneously fired neuronal activity, it could be used to indicate neuronal inhibition status.^[^ ^18^ ^]^ We first raised pMCH mice and injected the EGFP AAV virus (PT‐4250 ScAAV‐hSyn‐DIO‐EGFP‐WPRSaav2/9) into the LH region. Our observations revealed a notable increase in the amplitude of sIPSCs within the osteoporotic group compared to the control group. This difference between the two groups was found to be statistically significant (Figure 2D).

Since the hypothalamus is the biggest neuroendocrine organ where hormones disseminate to circulation, we tested whether MCH was released into circulation upon electrical stimulation. To precisely manipulate MCH neurons in LH, we introduced pMCH‐Cre mice, which were specifically engineered to target MCH neurons in LH. The use of pMCH‐Cre mice provides a controlled model to observe the effects of stimulating or inhibiting MCH neurons on hormone dissemination into circulation. To precisely manipulate MCH neurons in LH in pMCH‐Cre mice, we used chemogenetics technique by which neurons can be stimulated or inhibited for extended periods of time upon chemical signals.^[^ ^19^ ^]^ After injection of adenovirus (AAV) in specific area of interest, neurons are allowed to express designer receptors exclusively activated or inhibited by designer drugs (DREADDs), which are activated by clozapine‐N‐oxide (CNO).^[^ ^20^ ^]^ The viruses used for activation, inhibition, control were rAAV‐hSyn‐hM3D(Gq)‐EGFP‐WPRE‐hGH polyA, rAAV‐hSyn‐hM4D(Gi)‐EGFP‐WPRE‐hGH polyA and rAAV‐hSyn‐EGFP‐WPRE‐hGH polyA, respectively (Figure 2E). We found a burst of MCH released into circulation ≈2 h after chemogenetic stimulation of MCH neurons in LH with the highest plasma concentration at 200 pg mL^−1^. However, chemogenetic inhibition and control group did not show significant changes of MCH in circulation (Figure 2F). This result indicated that MCH was released into circulation upon electrical stimulation of the MCH neuron in LH.

To further delineate the action of MCH in bone metabolism in the periphery, we quantified the MCH concentrations in the cerebrospinal fluid (CSF) of osteoporotic mice. Our data indicate that osteoporotic mice exhibited significantly reduced MCH levels in the CSF when compared to control mice, with levels dropping from ≈90 to ≈40 pg mL^−1^ (Figure 2G). This reduction in CSF MCH levels implies a potential association between diminished MCH activity and the development of osteoporosis. In parallel, we assessed the plasma MCH levels in osteoporotic patients and observed a marked decrease in MCH concentrations relative to healthy controls, with levels decreasing from ≈30 to ≈20 pg mL^−1^ (Figure 2H). This clinical observation reinforces the hypothesis that lower MCH activity is linked to decreased bone density and the progression of osteoporosis.

Taken together, these results suggest that the activity of MCH neurons in LH correlate with bone density, and MCH is released into circulation once these neurons are stimulated.

### 2.3 Activating MCH Neurons in LH Induces Osteogenesis and Protect Against Skeletal Senescence

**Figure 3:**
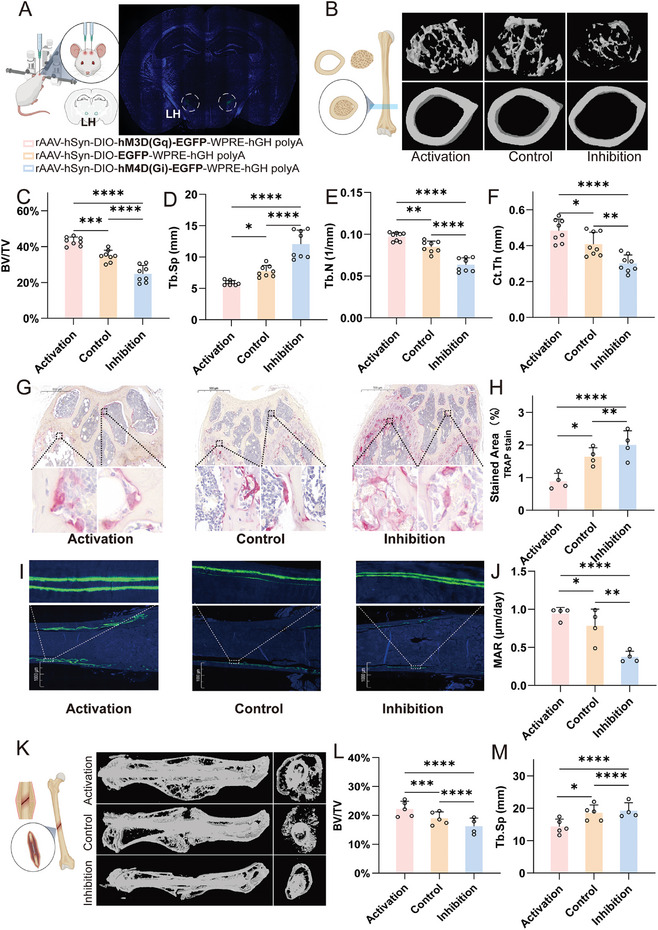
Activating MCH neurons in LH induces osteogenesis. A) schematic of the injection method for chemogenetic virus in LH. B) Representative images of Micro‐CT 3D reconstruction of the trabecular and cortical bone of activation, inhibition, and control group in OSTEOPOROTIC mice. C–F) Micro‐CT analysis of BV/TV, Tb.Sp, Tb.N and Ct.Th of the activation, inhibition, and control group in OSTEOPOROTIC mice. (n = 8). G,H) Representative image and statistical analysis of the TRAP staining of activation, inhibition, and control group in OSTEOPOROTIC mice (n = 4). I,J) Representative image and statistical analysis of the calcein labeling of activation, inhibition, and control group in OSTEOPOROTIC mice (n = 4). K) Representative images of Micro‐CT 3D reconstruction of the bone callus of activation, inhibition, and control group in femoral fracture mice. L,M) Micro‐CT analysis of BV/TV and Tb.Sp of the activation, inhibition, and control group in femoral fracture mice. (n = 5). ^*^ *p* < 0.05, ^**^ *p* < 0.01, ^***^ *p* < 0.001, ^****^ *p* < 0.0001; 1‐way ANOVA with Bonferroni's correction for multiple comparisons.

To test the role of MCH neurons in LH in osteogenesis and bone regeneration, we performed 2 type of animal models, including age induced osteoporosis and aged fracture repair. In osteoporosis model, we chemogenetically activated MCH neurons in LH when age induced osteoporosis was seen in pMCH‐Cre mice in 18 months old (**Figure** **3**A). Micro‐CT analysis was employed to investigate both the trabecular bone and cortical bone of the distal femur, as illustrated in Figure 3B. The ratio of trabecular bone volume to tissue volume (BV/TV) was significantly higher in the activation group (20%) compared with control group (15%) and inhibition group (10%) (Figure 3C). The trabecular number (Tb. N), trabecular separation (Tb.Sp), and connectivity (Conn. Dn) were partially restored following chemogenetic activation compared with control group and inhibition group (Figure 3D–F). To demonstrate the resorption and formation status after MCH neurons in LH activation, we did Tartrate‐resistant acid phosphatase (TRAP) staining and in vivo calcein labeling respectively. TRAP staining was performed to evaluate bone resorption, indicating less TRAP positive cells of trabecular bone in the activation group compared with the control group and inhibition group (Figure 3G,H). In vivo calcein labeling demonstrated that rates of new bone formation were higher in the activation group compared with the control group and inhibition group (Figure 3I,J).

In senile fracture repair model, we used the three‐point bending technique to induce a controlled fracture followed by intramedullary pinning for internal fixation.^[^ ^21^ ^]^ We aimed to use the fracture model to test the new bone formation and fracture repair followed by stimulation of MCH neuron in LH. First, we used micro‐CT to analyze the bone callus formation at the fracture healing site 4 weeks after fracture. Better fracture repair and larger bone callus formation could be seen in MCH activation group compared with control and inhibition groups (Figure 3K). Consistently with age induced osteoporosis model, significantly higher BV/TV and lower Tb. Sp were seen following chemogenetic activation compared with control group and inhibition group (Figure 3L,M), indicating better quality of bone callus formation. Taken together, these 2 animal models demonstrate that activation of MCH neurons in LH robustly induce osteogenesis and protect against skeletal senescence.

### 2.4 Inhibiting MCH Neurons in LH Induces BMSC Senescence

**Figure 4:**
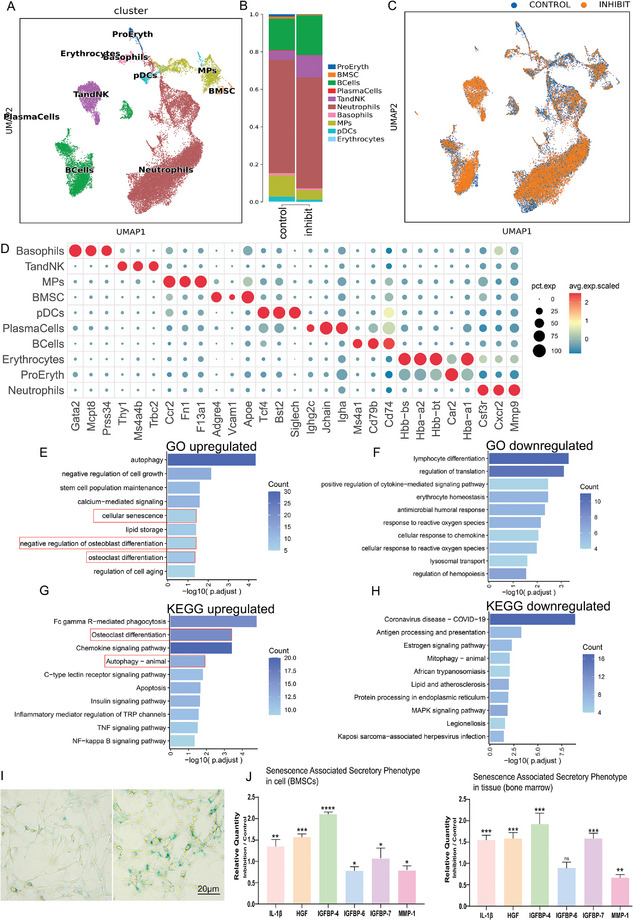
Inhibiting MCH neurons in LH retards BMSC activity. A) Visualization of bone marrow cells with identification of 10 clusters by single cell sequencing. B) Subpopulation proportion of 10 clusters with or without chemogenetic inhibition of MCH neurons in LH. C) Differentiation of cell clusters with our without chemogenetic inhibition of MCH neurons in LH. D) Dot plots showing average expression of known markers in indicated cell clusters. The dot size represents percent of cells expressing the genes in each cluster. The expression intensity of markers is shown. E,F) Representative pathways of upregulated (E) and downregulated (F) genes in the MCH neuron inhibition group compared to the control group based on biological pathway classification entries in the GO database. G,H) Representative pathways of upregulated (G) and downregulated (H) genes in the MCH neuron inhibition group compared to the control group based on biological pathway classification entries in the KEGG database. (I) Representative β‐Gal staining in the MCH neuron inhibition group compared to the control group. I,J) SASP from BMSC (left) and bone marrow (right) in the MCH neuron inhibition group compared to the control group.

To further elucidate the primary cell types and signaling pathways involved, we employed chemogenetic inhibition of MCH neurons in LH and subsequently conducted single‐cell RNA sequencing (scRNA‐seq). scRNA‐seq, a widely acclaimed method, enables the analysis of gene expression at the individual cell level, offering a detailed view of cellular diversity within populations. After quality control procedures, which included the removal of damaged or dead cells and potential cell doublets, we successfully retained a total of 18271 cell transcriptomes from both the MCH neuron inhibition group and the control group for further analysis. We utilized Uniform Manifold Approximation and Projection (UMAP) for visualizing transcriptional heterogeneity, which identified 10 distinct cellular clusters, including bone marrow stromal cells (BMSCs), as shown in **Figure** **4**A. Notably, we observed a reduction in the proportion of BMSCs following the chemogenetic inhibition of MCH neurons in the LH (Figure 4B). This alteration in cellular cluster distribution, with and without the chemogenetic inhibition of MCH neurons in the LH, is depicted in Figure 4C.

To delve deeper into the biological processes associated with BMSCs, we conducted a gene ontology (GO) analysis focusing on genes differentially expressed in BMSCs. The GO analysis indicated an upregulation in processes such as cellular senescence, negative regulation of osteoblast differentiation, and osteoclast differentiation (Figure 4E), while processes related to lymphocyte differentiation were downregulated (Figure 4F).

Furthermore, we performed a Kyoto Encyclopedia of Genes and Genomes (KEGG) pathway analysis to explore the functional pathways in BMSCs under the influence of chemogenetic inhibition of MCH neurons in the LH, compared to the control group. This analysis revealed an upregulation in several pathways associated with bone resorption, including osteoclast differentiation and autophagy, in the MCH neuron inhibition group (Figure 4G). Conversely, pathways such as the estrogen signaling pathway and mitophagy were found to be downregulated (Figure 4H). Upon further analysis of isolated Bone Marrow Stromal Cells (BMSCs), we noticed a substantial increase in β‐galactosidase (β‐gal) activity in the group where MCH neurons were inhibited (Figure 4I). β‐gal ’s activity is commonly used as a biomarker for cellular senescence, indicating aging cells. Additionally, there was a significant upregulation in the expression of the senescence‐associated secretory phenotype (SASP) in the inhibition group compared to the control group (Figure 4J). SASP is characterized by the secretion of inflammatory cytokines, growth factors, and proteases, and is known to contribute to the aging process and age‐related diseases.

Collectively, these scRNA‐seq findings suggest that the chemogenetic inhibition of MCH neurons in the LH specifically attenuates the activity of BMSCs, highlighting the intricate interplay between neuronal signaling and bone marrow cellular dynamics.

### 2.5 MCH Induces Osteogenic Differentiation in BMSC via PKA Signaling

**Figure 5:**
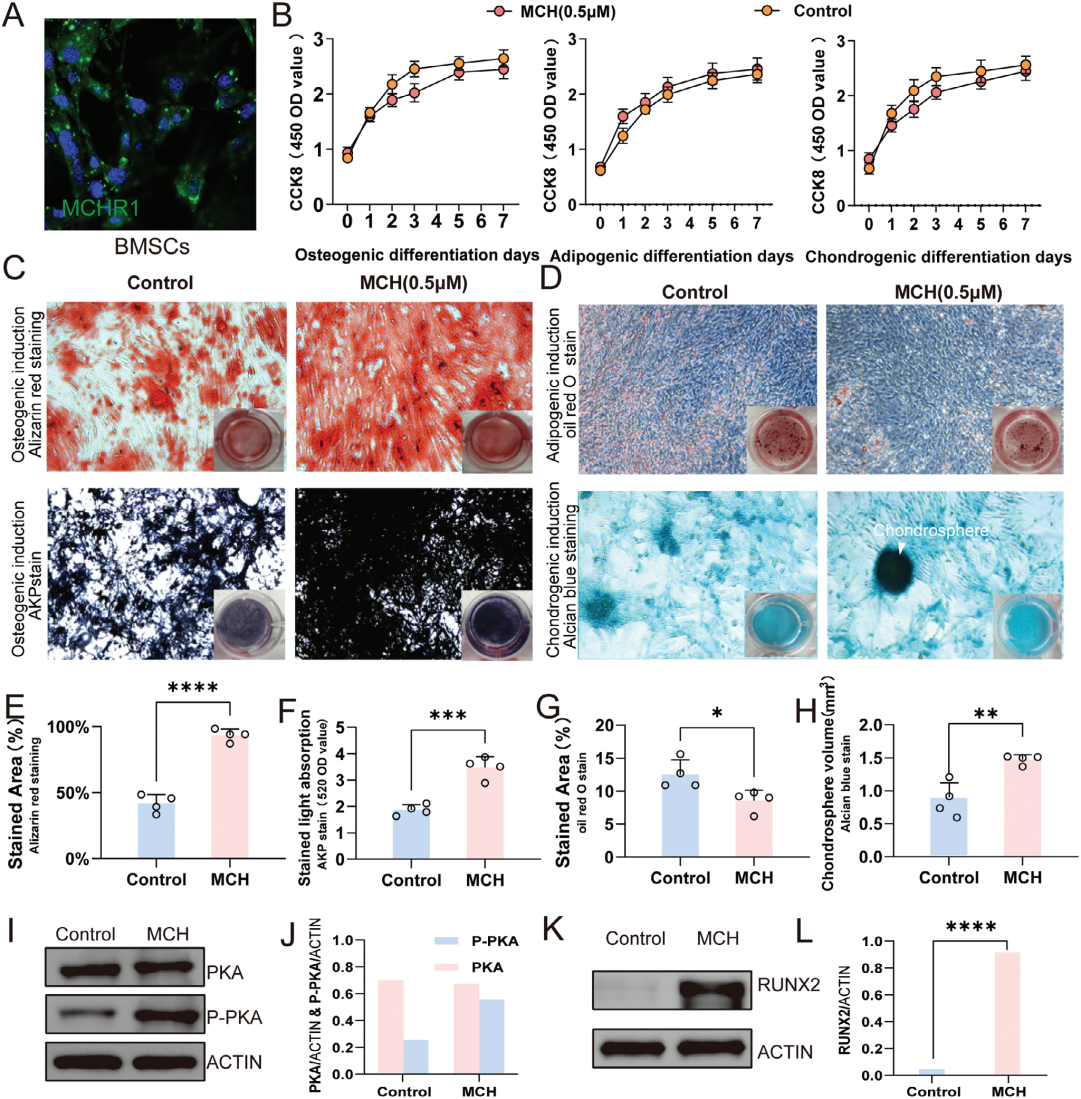
MCH induces osteogenic differentiation in BMSC via PKA signaling. A) Representative image showing MCHR1 is expressed on BMSCs. B) Plots showing CCK8 activities with or without MCH administration in osteogenic, adipogenic, and chondrogenic differentiation. C) Representative image showing Alizarin red (upper) and AKP (lower) positive cells in osteogenic induction of BMSCs. D) Representative image showing oil red O (upper) and Alcian blue (lower) positive cells in adipogenic and chondrogenic induction of BMSCs. E,F) The statistical analysis of Alizarin red and AKP positive cells in osteogenic induction of BMSCs (n = 4). G) The statistical analysis of oil red O positive cells in adipogenic induction of BMSCs (n = 4). H, The statistical analysis of Alcian blue positive cells in chondrogenic induction of BMSCs (n = 4). I and J, Representative image and statistical analysis of PKA signaling with or without MCH administration in BMSCs. K,L) Representative image and statistical analysis of PKC signaling with or without MCH administration in BMSCs. ^*^ *p* < 0.05, ^**^ *p* < 0.01, ^***^ *p* < 0.001, ^****^ *p* < 0.0001; student *t*‐test.

Since we demonstrated MCH neurons in LH release MCH into circulation upon chemogenetic stimulation, we hypothesize that MCH locally induces osteogenesis through activating MCH receptors once it is released into bone area. First, we tested the expression of MCH receptors on different cell types including BMSC and osteoclasts. By immunostaining, we found MCH receptor 1 (MCHR1) was specifically expressed on BMSCs (**Figure** **5**A). To test whether MCH administration influences BMSCs proliferation, we performed osteogenic, adipogenic, and chondrogenic differentiation with or without MCH. Using CCK8 as an indicator, we found 0.5µm MCH administration have slight influence on osteogenic, adipogenic, and chondrogenic differentiation of BMSCs (Figure 5B).

Subsequently, we tested the effect of MCH on osteogenic differentiation of BMSCs by Alizarin red and AKP staining (Figure 5C). The osteogenic potential of BMSCs treated with MCH was significantly higher than control group, as evidenced by elevated calcium nodule deposition in the Alizarin Red (Figure 5E) and AKP staining (Figure 5F). Moreover, to test the adipogenic and chondrogenic potential of BMSCs with or without MCH administration, we performed oil red O and Alcian blue staning respectively. We found a decrease in the adipogenic potential (Figure 5G) and an increase in the chondrongenic potential (Figure 5H) of BMSCs treated with MCH compared to the control group. To elucidate the signal transduction pathway of MCH on BMSC, we tested PKA pathway following previous studies.^[^ ^22^ ^]^ The western blot analysis showed that phosphorylated PKA was significantly upregulated following the MCH administration (Figure 5I,J). We tested the master osteogenic transcriptional factor runx2 signaling was also upregulated with MCH administration (Figure 5K,L). Since the majority of osteogenic pathways were related to PKA signaling, we focused on the PKA signaling in the following study. Taken together, these results collectively suggested that MCH induced osteogenic differentiation of BMSCs through MCHR1 via PKA signaling.

### 2.6 MCH Activates Osteogenic and Inhibit Senescence Pathways in BMSCs

**Figure 6:**
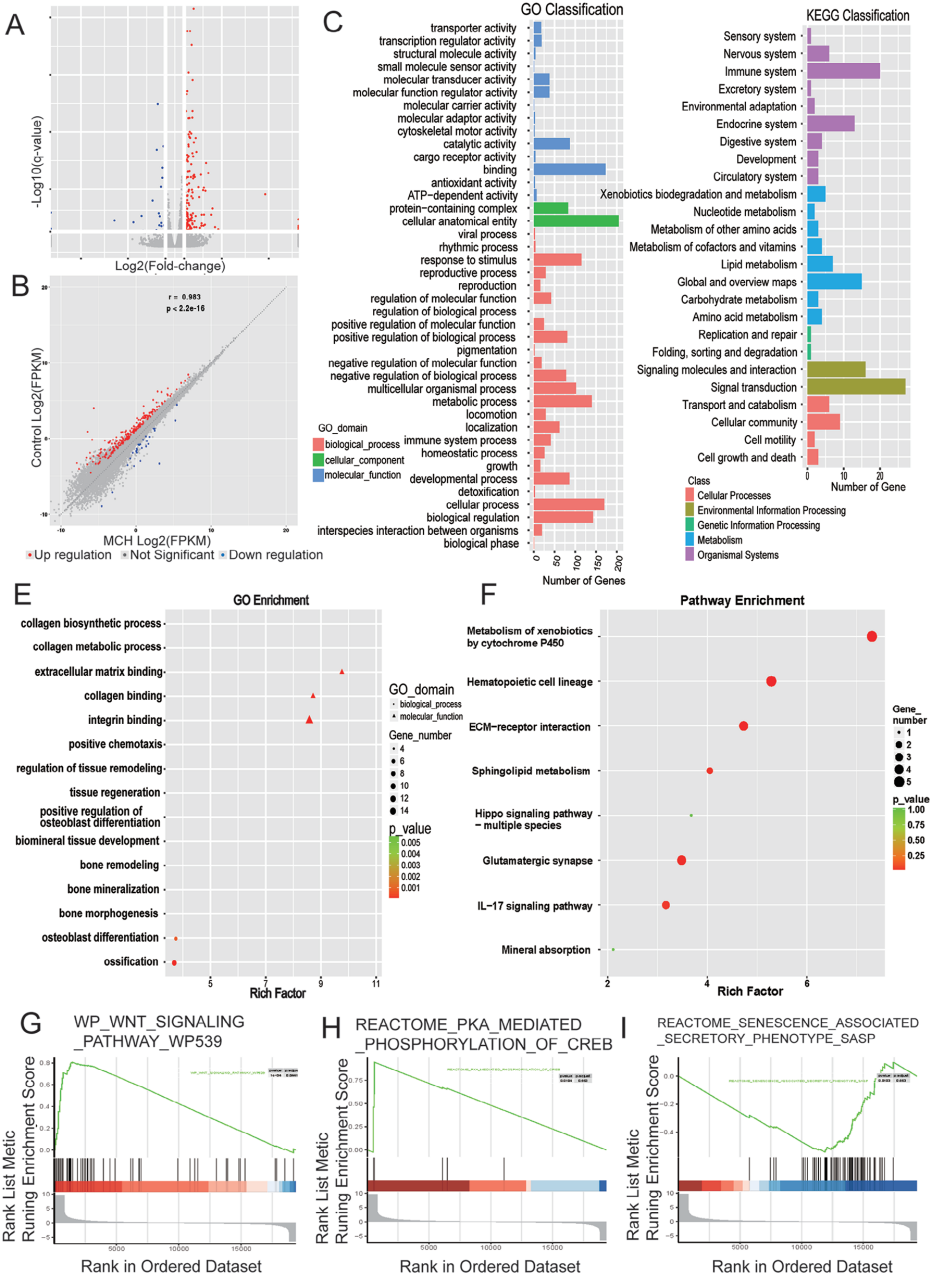
MCH activates osteogenic and inhibits senescence pathways in BMSCs. A) Volcano plot depicting the distribution of differentially expressed genes in the MCH group compared to the control group in BMSCs. B) Correlation of the differentially expressed genes in the MCH group compared to the control group in BMSCs. C,D) Representative GO classification and KEGG pathways of differentially expressed genes in the MCH group compared to the control group in BMSCs. E,F) GO and KEGG enrichment analysis of the MCH group compared to the control group in BMSCs. G‐I) GSEA analysis of Wnt, PKA and SASP pathways in the MCH group compared to the control group in BMSCs.

To explore the molecular processes driving the osteogenic differentiation of BMSCs upon MCH administration, we employed advanced high‐throughput mRNA sequencing. For this purpose, BMSCs, both treated and untreated with MCH, were subjected to mRNA sequencing following a 9‐day period of osteogenic induction. The subsequent quantitative analysis of gene expression levels revealed notable differences between the two groups. A total of 212 genes were found to be upregulated, while 31 genes exhibited downregulation in the MCH‐treated BMSCs. Noteworthy among the upregulated genes were alkaline phosphatase and integrin family proteins, both of which showed significant elevation. These genes, known for their roles in bone metabolism and cell adhesion respectively, underscore the osteogenic potential of MCH. This differential gene expression was eloquently illustrated through volcano and waterfall plots (**Figure** **6**A,B).

Furthermore, Gene Ontology (GO) enrichment analysis delineated a series of bone formation pathways significantly influenced by MCH treatment. These pathways included skeletal system development, ossification, integrin complex formation, bone mineralization, and Wnt‐protein binding. In contrast, pathways like negative regulation of RNA Polymerase II transcriptional activity and apoptosis were significantly subdued in the MCH group (Figure 6C). Additional GO analysis highlighted the enrichment of processes like collagen biosynthesis, tissue regeneration, bone remodeling, and ossification (Figure 6E). Complementing the GO analysis, Kyoto Encyclopedia of Genes and Genomes (KEGG) pathway enrichment analysis, conducted using the latest database, categorized the differentially expressed genes into representative biological pathways. Predominantly, pathways related to bone formation such as TGF‐beta signaling, Wnt signaling, Hippo signaling, and parathyroid hormone signaling were markedly upregulated in the MCH‐treated group. Conversely, pathways associated with cellular senescence and osteoclast differentiation were found to be downregulated (Figure 6D). The KEGG analysis further corroborated the enrichment of the ECM receptor interaction and Hippo signaling pathways (Figure 6F).

To refine our understanding and eliminate potential ambiguities from the separate analyses of up‐ and down‐regulated genes, we selected three pathways closely associated with bone formation for a more focused Gene Set Enrichment Analysis (GSEA). This analysis reinforced our previous findings, highlighting a significant upregulation of the Wnt signaling pathway and PKA signaling, while SASP signaling was notably downregulated in the MCH‐treated group (Figure 6G–I).

In summary, our comprehensive study elucidates that MCH administration triggers a cascade of biological pathways, fostering osteogenic differentiation in BMSCs. This discovery opens new vistas in understanding the molecular mechanisms underlying bone formation and regeneration.

### 2.7 Activating MCHR1 Alleviates Skeletal Senescence in Mice

**Figure 7:**
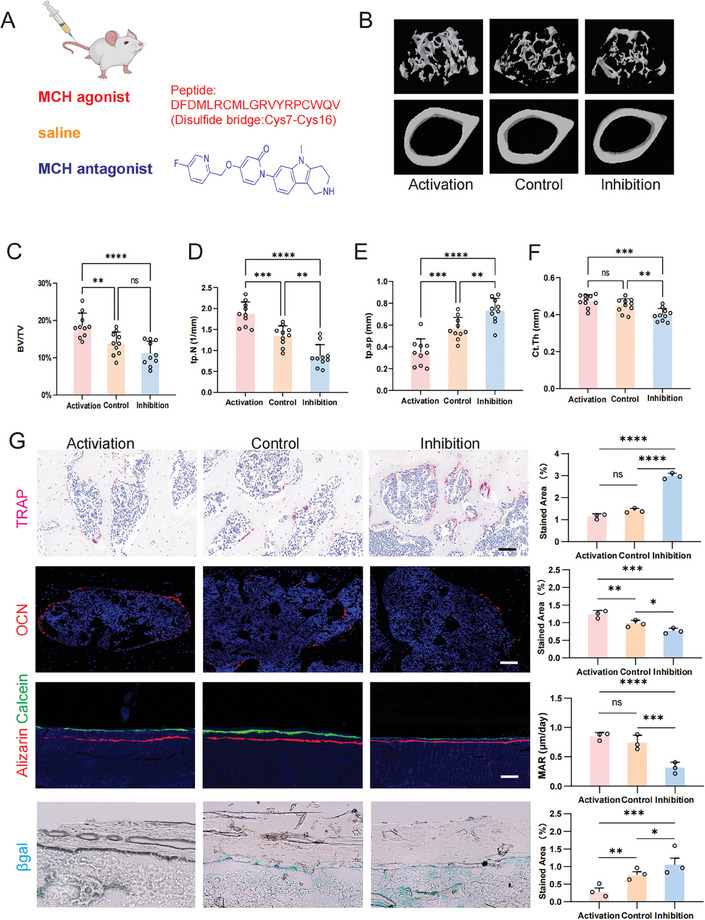
Activating MCHR1 induces osteogenesis and alleviates osteoporosis in mice. A) Schematic of the tail vein injection of MCHR1 agonist and antagonist in age‐induced osteoporotic mice. B) Representative images of Micro‐CT 3D reconstruction of the trabecular and cortical bone of MCHR1 activation, inhibition, and control group in osteoporotic mice. C‐F, Micro‐CT analysis of BV/TV, TbN, Tb.N, Tb.Sp and Ct.Th of the MCHR1 activation, inhibition, and control group. (n = 10). G and H, Representative image and statistical analysis of the TRAP‐positive cells of MCHR1 activation, inhibition, and control group in osteoporotic mice (n = 3). I and J, Representative image and statistical analysis of the OCN positive cells of MCHR1 activation, inhibition, and control group in osteoporotic mice (n = 3). K,L) Representative image and statistical analysis of the calcein labeling of activation, inhibition, and control group in osteoporotic mice (n = 3). M and N, Representative image and statistical analysis of the β‐gal staining of activation, inhibition and control group in osteoporotic mice (n = 3).^*^ *p* < 0.05, ^**^ *p* < 0.01, ^***^ *p* < 0.001, ^****^ *p* < 0.0001; 1‐way ANOVA with Bonferroni's correction for multiple comparisons.

**Figure 8:**
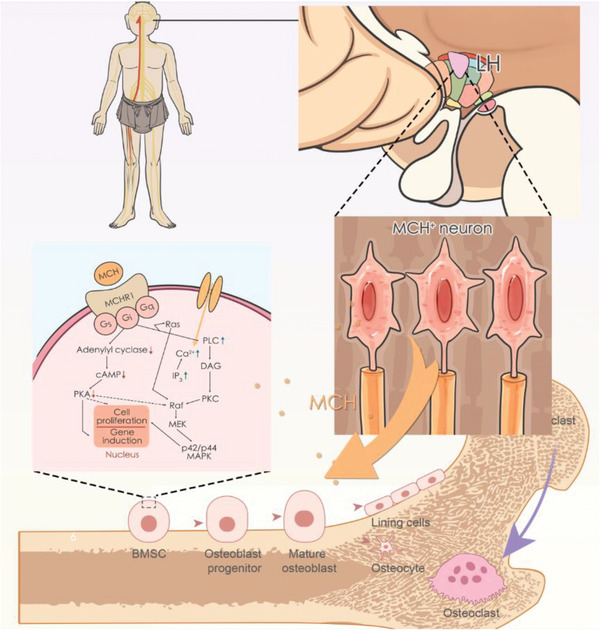
Working model of neuroendocrine circuit in LH for bone mass regulation through MCH signaling.

To investigate the effect and therapeutic potential of MCHR1 signaling in skeletal senescence, we intravenously delivered both MCHR1 agonist (MCH TFA) and antagonist (ALB‐127158a) or vehicle (saline) for age induced osteoporosis in mice (**Figure** **7**A). With the development of osteoporosis, micro‐CT analysis of distal femurs revealed significant bone loss in consistent with previous results. Notably, mice treated with MCH agonist, exhibited markedly increased bone mass compared to vehicle treated osteoporotic mice (Figure 7B). This increase was evident in terms of the BV/TV (Figure 7C), Tb.N (Figure 7D), and decreased Tb.Sp (Figure 7E). However, there was no difference in Ct.Th between the MCH agonist and vehicle group (Figure 7F). Consistently, the mice treated with MCH antagonist showed significant reduction in Tb.N, Ct.Th (Figure 7D,F) and increase in Tb.Sp (Figure 7E). These findings suggest that the MCH agonism significantly attenuates bone loss in mouse model of osteoporosis. To determine whether MCH agonism activates bone remodeling, we assessed markers of bone formation and resorption. Compared to the control mice, MCH agonist treated mice exhibited a significant reduction of TRAP positive cells, indicating that MCH agonism decreased osteoclast activity (Figure 7G,H). Consistently, an increased number of OCN positive cells, as evidenced by OCN staining on trabecular bone in MCH agonist treated mice compared to the vehicle group (Figure 7I,J). Moreover, in vivo calcein labeling showed increased MAR in MCH agonist group compared with the control group (Figure 7K,L). Meanwhile, we noted the decreased OCN level, decreased MAR, and increased TRAP positive cells in the MCH antagonism treated mice, indicating lower bone formation and elevated bone resorption compared to the control group. To further substantiate the therapeutic efficacy of MCHR1 signaling in mitigating skeletal senescence, we conducted an analysis of β‐gal staining. Consistent with our prior observations, the administration of the MCH agonist resulted in a pronounced reduction in senescence‐associated β‐gal markers when compared to the vehicle group, as depicted in **Figure** **8**M. This was further quantified through a significant decrease in the prevalence of β‐gal positive cells within both the bone marrow and trabecular regions in mice receiving MCH agonist therapy (Figure 8M,N). Conversely, the administration of the MCH antagonist led to a notable increase in β‐gal positive cells, indicative of accelerated senescence within the bone tissues. Collectively, these results provide compelling evidence that MCH agonism attenuates skeletal senescence in mice by activating osteogenesis and suppressing osteoclastogenesis.

## 3 Discussion

Bone is a specialized organ with various functions. Through getting mechanical strength by biological mineralization, bone serves mechanical functions such as providing rigidity, shape, protection of the body structures and facilitating movement. Despite supporting functions, bone undergoes constant remodeling to maintain metabolic homeostasis and keep regeneration.^[^ ^23^ ^]^ The overall mechanical properties of the bone, including its rate of turnover, collagen matrix, size, structure, geometry, and density, are critical in determining its strength and resilience. Any defects in these parameters can result in various skeletal disorders, such as osteoporosis, osteopetrosis, Paget's disease, and osteogenesis imperfecta.^[^ ^24^ ^]^

It has long been hypothesized that both central and peripheral nervous system are responsible for controlling bone metabolism and disorders. Central neuropeptides are known to play crucial roles in the regulation of bone metabolism.^[^ ^15^, ^25^ ^]^ For instance, leptin inhibits osteogenesis through activating the sympathetic nervous system,^[^ ^4^ ^]^ and orexin maintains skeletal metabolic homeostasis with a dual role in bone mass regulation.^[^ ^26^ ^]^ For centuries, the existence of local nerve fibers supplying and regulating the activity of bone metabolism has been recognized.^[^ ^3^ ^]^ Meanwhile, there is increasing evidence showing that different types of neurons play distinct roles in the regulation of bone metabolism and homeostasis. Sensory nerves play a critical role in detecting mechanical stress and strain on bones, which is necessary for bone remodeling and adaptation.^[^ ^6b^ ^]^ Sympathetic nerves regulate bone resorption and formation by releasing neurotransmitters such as norepinephrine and neuropeptide Y.^[^ ^6^, ^15^ ^]^ Although these molecular and genetic studies have provided valuable insights into the neuropeptides that regulate bone mass, studies on the central neural regulation of bone metabolism and skeletal senescence at the neuroendocrinological level have been very limited.

Skeletal senescence is a complex process leading to reduced bone mass and increased fragility, often resulting in conditions like osteoporosis.^[^ ^27^ ^]^ This process is driven by changes at the cellular level, particularly in osteoblasts and osteoclasts responsible for bone formation and resorption. As these cells age, there's a decline in bone formation and an increase in bone resorption, contributing to overall bone mass loss.^[^ ^28^ ^]^ This cellular aging is influenced by systemic factors such as hormonal changes and local factors like oxidative stress, which impair bone regeneration and increase fragility.^[^ ^29^ ^]^ Additionally, skeletal aging involves alterations in bone architecture and quality, significantly impacting the risk of fractures in the elderly. Key molecular pathways, such as p53/p21 and p16INK4a/Rb, are activated in response to stressors like DNA damage, leading to cellular senescence.^[^ ^30^ ^]^ Genetic factors also play a crucial role in bone aging, with specific genes and transcription factors regulating mesenchymal stem cell functions and influencing bone density and quality during aging.^[^ ^31^ ^]^ Moreover, the interaction between the immune system and bone tissue becomes increasingly important in the context of aging. Senescent immune cells release factors that contribute to the acceleration of bone aging, underscoring the complex relationship between these systems.^[^ ^32^ ^]^ However, whether targeting neuroendocrinological way is effective for skeletal senescence is still largely unknown.

As an anabolic hormone, MCH promotes increased food intake and energy storage,^[^ ^33^ ^]^ which supports its role in bone formation by enhancing osteoblast activity and reducing osteoclast activity. This contrasts with leptin, which inhibits bone osteogenesis and promotes energy expenditure. Unlike MCH, orexin positively influences bone formation and is crucial for maintaining bone health.^[^ ^26^ ^]^ Moreover, a Chinese traditional drug called Zuogui Wan improves trabecular bone microarchitecture in ovariectomy‐induced osteoporosis rats by regulating orexin‐A and orexin receptor.^[^ ^34^ ^]^ MCH is known to be released from the hypothalamus into the circulation, exerting its effects both centrally and peripherally. Studies have demonstrated that MCH may cross the blood‐brain barrier (BBB) and enter the CSF before making its way into the bloodstream.^[^ ^35^ ^]^ This dual release mechanism allows MCH to impact a variety of physiological processes, including energy balance, feeding behavior, and bone metabolism. One primary mechanism through which MCH enters the circulation involves tanycytes, specialized glial cells in the hypothalamus. MCH may utilize receptor‐mediated transcytosis to cross the endothelial cells of the BBB. This process involves MCH binding to specific receptors, being internalized, and then released on the other side, allowing it to enter the CSF and subsequently the bloodstream.^[^ ^36^ ^]^ Furthermore, MCH can diffuse through circumventricular organs (CVOs), regions of the brain with naturally more permeable BBBs, facilitating direct exchange between the blood and the brain.

MCH exerts dual actions through its neuroendocrine release into the circulation and its interactions with the sympathetic nervous system (SNS). Centrally, MCH influences feeding behavior, energy expenditure, and adiposity by acting on MCH receptors in the hypothalamus and other brain regions. Peripherally, MCH affects various metabolic processes, including bone formation, by interacting with receptors in peripheral tissues. The neuroendocrine action of MCH involves its release into the bloodstream, where it can interact with distant organs. Simultaneously, MCH can modulate the SNS, affecting bone metabolism by altering sympathetic tone. This dual mechanism allows MCH to coordinate complex physiological responses. Recent research by Saito et al.^[^ ^37^ ^]^ supports these dual roles, demonstrating how MCH can influence both central and peripheral systems through its actions on the circulation and the SNS.^[^ ^12^ ^]^

Here we identified a neuroendocrinological circuit underlying skeletal interoception to regulate bone metabolism. The circuit is based on several lines of evidence. First, we used virus‐based tracing strategy to identify MCH neurons but not orexin neurons in LH are trans‐synaptically connected to the bone. Second, we found the electrical activity of MCH neurons in LH were highly correlated with bone metabolism status. To investigate whether MCH was secreted into circulation upon stimulation, we used chemogenetic tools to confirm that MCH was secreted into plasma upon electrical stimulation by MCH neurons in LH. Third, to further investigate the effect of MCH neurons in LH in bone mass regulation, we used chemogenetic viruses and transgenic mice model to manipulate this specific group of neurons. Chemogenetic stimulation of MCH neurons in LH results in robust osteogenesis. Fourth, to elucidate the specific cell type responsible for MCH action of osteogenesis, we performed scRNA‐seq. The results suggested that BMSCs were specifically influenced by chemogenetic inhibition of MCH neurons in LH. Fifthly, we performed in vitro experiments demonstrate that MCHR1 is expressed BMSCs and MCH induces osteogenesis through MCHR1 activation of PKA pathway. Lastly, we test that MCHR1 agonist is capable of attenuating osteoporosis in mice.

Based on our results, we propose a working model where MCH neurons in LH is the center of skeletal interoception and neuroendocrine regulation of bone mass (Figure 8). First, the MCH neurons trans‐synaptically receive the signal from bone through sensory nerve for bone density surveillance. At the same time, when stimulated by sensory signals, the MCH neurons in LH secret MCH into circulation to accelerate BMSC osteogenic differentiation through MCHR1 via PKA signaling. We found a central peptide MCH in LH has been functionally demonstrated to control skeletal interoception and locally induce osteogenesis upon ascending nervous signals. In the near future, if this mechanism is confirmed in human, targeting MCH neurons in LH shows therapeutic potential for various skeletal diseases.

## 4 Experimental Section

### Animals

The study utilized adult C57BL/6 mice, both male and female, obtained from the animal facility center at Central South University. The p‐MCH Cre mice were purchased from the Jackson Lab. The age‐induced osteoporotic mice were 18 months old, while the control mice were 8 weeks old. The mice were kept in a controlled environment with a temperature range of 22–25 °C, humidity between 40% and 60%, and subjected to a 12 h light/dark cycle. They had unrestricted access to food and water. All animal care and experimental procedures were approved by the Research Committee of Xiangya Hospital, Central South University. The study included an equal number of male and female mice.

### Tail Vein Injection

For the tail vein injections, MCH agonists (MCH, TFA) were administered at a dose of 18 mg kg^−1^ twice daily (bid) and were obtained from MedChemExpress (HY‐P1205A). MCH antagonists (ALB‐127158(a)) were administered at a dose of 10 mg kg^−1^ twice daily (bid) and were sourced from MedChemExpress (HY‐111398). Saline was used as a control and was administered at a volume of 0.1 ml per day. The total duration of these treatments was 30 days.

### Staining

For immunohistochemistry staining of bone samples, the samples were dehydrated using serial ethanol solutions (70%, 1 h × 2; 95%, 2 h × 2; 100%, 1 h × 2) and cleared with xylene (1 h × 2) before embedding them in paraffin (Surgipath Paraplast, Leica). Slices of 5–7 µm thickness were obtained using a microtome (RM2235, Leica) and transferred to adhesion glass slides. The slides were deparaffinized by washing with xylene (3 min × 2) and serial ethanol solutions (100%, 3 min × 2; 95%, 3 min × 2; 70%, 3 min × 2). The slices were incubated with primary antibodies, including anti‐MCH (1:100, Abcam), anti‐orexin (1:100, Abcam), anti‐VGAT (1:100, Abcam), anti‐EGFP (1:200, Sigma), anti‐c‐Fos (1:100, CST) at 4 °C overnight. After washing, the sections were labeled with fluorescent secondary antibodies. For secondary antibody staining, Alexa Fluor 488 or 594‐conjugated antibodies (1:100; Jackson ImmunoResearch, PA) were used. Nuclei were counterstained with DAPI, and the samples were then mounted for image acquisition.

### Western Blotting

Cells or tissues were homogenized in ice‐cold RIPA buffer (50 mM Tris‐HCl, pH 7.4, 150 mM NaCl, 1% NP‐40, 0.5% sodium deoxycholate, 0.1% SDS) with protease and phosphatase inhibitors. The homogenate was centrifuged at 12 000 rpm for 15 min at 4 °C, and the supernatant protein concentration was measured using a BCA assay. Equal amounts of protein (20–30 µg) were mixed with 4x Laemmli buffer, heated at 95 °C for 5 min, and loaded onto a 10% SDS‐PAGE gel. Electrophoresis was conducted at 100 V until the dye front reached the gel's bottom. Proteins were transferred to a PVDF or nitrocellulose membrane at 100 V for 60–90 min. Membranes were blocked in 5% non‐fat milk or BSA in TBST (Tris‐buffered saline with 0.1% Tween‐20) for 1 h at room temperature, then incubated overnight at 4 °C with primary antibodies in blocking buffer (anti‐PKA, PA5‐28160; anti‐pPKA, ZENBIO347334; anti‐Runx2, Abmart T55395F;). After three TBST washes, membranes were incubated with HRP‐conjugated secondary antibodies for 1 h at room temperature. Bands were visualized using an enhanced chemiluminescence (ECL) system and captured on X‐ray film or a digital imaging system.

### Virus Based Tracing

To trace the anterograde pathways from the bone marrow to the lateral hypothalamus, HSV‐129 EGFP (BrainVTA, H01001) was used for multi‐site injections in the bone marrow of adult mice. The virus was administered at multiple injection sites, specifically targeting the femoral and tibial bone marrow cavities, with each site receiving 1 µL of virus. The total volume of virus injected per mouse was 10 µL. After the injections, the mice were allowed to survive for 7 days to enable adequate viral transport and expression of EGFP. Following this period, the mice were perfused, and tissues were collected from the brain and spinal cord for analysis.

### Chemogenetics

Adult mice aged 6–8 weeks were anesthetized intraperitoneally using sodium pentobarbital (100 mg kg^−1^) and gently secured to a stereotaxic frame (RWD Life Science, Shenzhen, China). A total volume of 200 nL of the virus suspension was loaded into a 1 ml‐Hamilton syringe fitted with a needle made of glass electrode. The AAVs used were rAAV‐hSyn‐DIO‐hM3D(Gq)‐EGFP‐WPRE‐hGH polyA, rAAV‐hSyn‐DIO‐EGFP‐WPRE‐hGH polyA rAAV‐hSyn‐DIO‐hM4D(Gi)‐EGFP‐WPRE‐hGH polyA, obtained from BrainVTA, Wuhan, China. The viral titer for all AAVs was maintained at 2.32‐5.72E+12 PFU/mL. Mice(C57BL/6‐Tg(Pmch‐cre)1Rck/J) carrying genes specifically expressing CRE in pMCH cells come from JAX lab, The injection was performed at a rate of 20 nL min^−1^ into specific target locations (−2.00 mm bregma, ±1.37 mm lateral to the midline, and at a depth of −4.65 mm). The surgical procedure adhered to the protocol outlined by Lowery and Majewska (reference 54). Following the surgery, a recovery period of at least 1 week was provided for the mice before commencing any experimental procedures. For chemogenetic activation and inhibition, clozapine‐N‐oxide (CNO) at a dosage of 1 mg kg^−1^ (MCE, 34233‐69‐7) was administered intraperitoneally three weeks after viral expression. The injections were repeated every 48 h for a duration of four weeks before serum and bone samples were collected for subsequent analysis. The AAV9 viruses were prepared in‐house, ensuring their quality and consistency.

### Calcein Labeling

In this investigation, mice were subjected to intraperitoneal injections of calcein at doses of either 20 mg kg^−1^, which were dissolved in a 2% sodium bicarbonate solution. Calcein labeling was conducted over a period of 5 days preceding the sacrifice of the mice. The femur bones were fixed in a solution composed of 70% ethanol and subsequently embedded in methylmethacrylate for sectioning. Analysis of the bone surface and quantification of the bone formation rate (BFR) per unit of bone surface (BS). Furthermore, the mineral apposition rate (MAR) was determined by measuring the distance between the midpoints of two labels and dividing it by the time interval between the midpoints.

### Micro‐CT

The femurs of mice were harvested and fixed in 4% paraformaldehyde before undergoing micro‐CT scanning with a SkyScan 1076 system. Scanning parameters included an isotropic voxel size of 11.53 mm, a voltage of 48 kV, a current of 179 mA, and an exposure time of 1800 ms. 3D reconstructions of the scans were performed using SkyScan NRecon software with a voxel size of 8.66 mm. The datasets were reoriented using SkyScan DataViewer, and morphological parameters were quantified using CTAn software.

For trabecular bone analysis, a region of interest (ROI) was manually delineated from 2D image slices, specifically 0.1–0.9 mm distal to the proximal tibia growth plate, excluding the cortical area. Binarization was conducted using a global thresholding approach (gray level range 70–255) to differentiate mineralized tissue. A Gaussian filter with a radius of 1 was applied for 3D reconstruction. Quantitative analysis covered all bone areas within the ROI. Morphometric parameters included total volume (TV), bone volume (BV), bone fraction (BV/TV), trabecular thickness (Tb. Th), trabecular number (Tb. N), trabecular separation (Tb. SP), and bone mineral density (BMD). BMD calibration was performed using hydroxyapatite phantoms with known densities.

For cortical bone analysis, the ROI was selected from 2D images, 6.5–7.2 mm proximal to the distal femur growth plate. A threshold range of 85–255 was used for mineralized tissue identification. A Gaussian filter radius of 1 was applied for 3D reconstruction. Quantitative analysis included bone volume (BV) and bone mineral density (BMD) within the ROI.

### Electrophysiology

In this experiment, mice underwent deep anesthesia followed by transcardial perfusion with an ice‐cold cutting solution enriched with 95% oxygen and 5% carbon dioxide, using N‐methyl‐D‐glucamine (NMDG) as the primary component. Post‐perfusion, the brain was quickly removed and submerged in the same NMDG‐based solution at low temperature. Coronal brain slices of approximately 300 µm were prepared from the lateral hypothalamus (LH) using a vibratome. These slices were initially recovered in the cutting solution at a slightly higher temperature and then transferred to oxygenated artificial cerebrospinal fluid (ACSF) for further preparation before electrophysiological recordings.

For recordings, the slices were continuously perfused with oxygenated ACSF and visualized using an upright microscope with infrared optics. MCH neurons in the LH were identified by their fluorescence and morphology through EGFP expression, marking them as MCH‐positive. To study spontaneous inhibitory postsynaptic currents (sIPSCs), the intracellular recording solution contained cesium chloride (CsCl), isolating GABAergic currents by blocking AMPA and NMDA receptors. The holding potential was set at 0 mV. Neuronal stimulation was not applied, allowing for the observation of spontaneous events.

Recorded signals were amplified, filtered, and sampled using a MultiClamp 700B amplifier and appropriate software. Data validity was contingent on the series resistance remaining within a specific range. The number of neurons recorded varied and is detailed in the corresponding figure descriptions.

### BMSC Differentiation

For the induction of osteogenic differentiation, BMSCs were seeded at a density of 4 × 104 cells per well in 24‐well culture plates. After 24 h of incubation, the culture medium was replaced with osteogenic induction medium (OIM). As a negative control, cells were incubated in OIM without scaffold extracts but containing the same osteogenesis‐inducing factors. Throughout the differentiation process, the medium was refreshed every two days.

To induce differentiation into adipocyte‐like cells, BMSCs were cultured until reaching confluence. Two days after confluence, the medium was replaced with DMEM supplemented with 10% fetal bovine serum (FBS; Gibco), IBMX (0.5 mM; Sigma‐Aldrich), dexamethasone (0.25 µM; Sigma‐Aldrich), and bovine insulin (10 g/mL). After 72 h, the medium was further replaced with DMEM supplemented with 10% FBS and 10 µg/mL bovine insulin (Sigma‐Aldrich, I6634). The medium was refreshed every 2 days during the differentiation process. To assess the osteogenic differentiation potential of BMSCs, Alizarin Red S staining was performed. Additionally, oil red staining was used to evaluate the adipogenic differentiation potential.

### Single Cell Sequencing

The tissue dissociation and sample preparation involved several steps. Ascites samples were filtered through 40‐micron strainers and centrifuged to separate cells from the supernatant. The cell pellet was resuspended in PBS and treated with erythrocyte lysis buffer. Concurrently, fresh surgical tissues were preserved, washed, minced, and enzymatically digested. Post‐digestion, samples were centrifuged, and pellets resuspended in PBS, with cell viability assessed using Trypan Blue staining. For scRNA‐seq, single‐cell suspensions were processed using a microwell chip and barcoding beads for mRNA capture. Reverse transcription generated cDNA, followed by PCR amplification, cDNA fragmentation, and adapter ligation. scRNA‐seq libraries, prepared using a specific kit, were pooled and sequenced on an Illumina NovaSeq 6000 platform for 150 bp paired‐end reads. Raw sequencing reads were processed with CeleScope v1.5.2, using default parameters for gene expression profiling. Barcodes and UMIs were extracted and corrected from R1 reads, while R2 reads, trimmed of adapters and poly A tails, were aligned to the transcriptome using STAR and FeatureCounts. The resulting gene expression matrix was used for further analysis. Quality control, dimensionality reduction, and clustering analyses were conducted using Scanpy v1.8.1 in Python 3.7. The expression matrix underwent filtering to exclude cells with extreme gene or UMI counts and high mitochondrial content. Post‐filtering, normalization and logarithmic transformation were applied. The top 2000 variable genes were identified using the “seurat” method, and PCA was performed. Cells were clustered using the Louvain algorithm and visualized using UMAP. Then, the KEGG and GO analysis were performed.^[^ ^38^ ^]^

### RNA Sequencing

BMSCs treated with MCH or control were collected for RNA sequencing. After obtaining the raw sequencing data, quality control evaluation and comparative analysis of the sequencing data were performed. Sample expression levels were quantified, and differential expression analysis of genes and transcripts between the two groups was conducted using the R package Ballgown. Differentially expressed genes were identified using a threshold of a 0.5‐fold difference, a p‐value of ≤ 0.05, and a mean FPKM (reads per kilogram of transcript per million mapped reads) within the group ≥0.5. Fold change (FC) was calculated as FC = 2^(test_FPKM – control_FPKM)^, and the p‐value was determined using the F‐test for homogeneity of variance in the R package. For enrichment analysis of differentially expressed genes, the Entrez IDs of up‐regulated and down‐regulated genes were separately input into the R package cluster Profiler. The built‐in function enrich KEGG was used to perform KEGG pathway enrichment analysis. Additionally, all expressed genes' Entrez IDs and logFC data were input into the cluster Profiler package. The built‐in function gse KEGG was employed to conduct Gene Set Enrichment Analysis (GSEA) on each KEGG pathway identified in the previous step. The gseaplot2 function was used to visualize the GSEA results, where the p‐value was obtained through empirical phenotype‐based permutation tests to evaluate the nominal p‐value.

### Statistical Analysis

All experimental results were reported as mean ± standard error of the mean (SEM) with n indicating the number of animals. Statistical analysis was conducted using the appropriate unpaired Student's t‐test to compare control and treatment groups. For 3 or more group analysis, 1‐way ANOVA with Bonferroni's correction for multiple comparisons was used. The statistical software Prism version 8 (GraphPad Software, San Diego, CA, USA) was employed for all analyses. Statistical significance was determined when the probability (P) values were less than 0.05, denoting a significant difference between the groups.

## Conflict of Interest

The authors declare no conflict of interest.

## Author Contributions

B.G., Y.Z., and S.L. contributed equally to this work. J.Z. designed the experiments. B.G. and J.Z. conducted virus tracing experiments. B.G., Z.R., H.L., and C.W. conducted chemogenetics experiment. B.G., S.L., and X.C. conducted the electrophysiological experiments. B.G., Y.Z., and X.Y. performed osteoporosis and fracture mice models. B.G, Y.Z., and Z.R. performed cellular experiments including BMSC differentiation and collection of the samples for high throughout put sequencing. J.Z. and B.G. wrote the manuscript with the help of all authors.

## Data Availability

The data that support the findings of this study are available from the corresponding author upon reasonable request.
